# Troponin-Guided Coronary Computed Tomographic Angiography After Exclusion of Myocardial Infarction

**DOI:** 10.1016/j.jacc.2021.07.055

**Published:** 2021-10-05

**Authors:** Kuan Ken Lee, Anda Bularga, Rachel O’Brien, Amy V. Ferry, Dimitrios Doudesis, Takeshi Fujisawa, Shauna Kelly, Stacey Stewart, Ryan Wereski, Denise Cranley, Edwin J.R. van Beek, David J. Lowe, David E. Newby, Michelle C. Williams, Alasdair J. Gray, Nicholas L. Mills

**Affiliations:** a BHF Centre for Cardiovascular Science, University of Edinburgh, Edinburgh, United Kingdom; b Department of Emergency Medicine, Emergency Medicine Research Group, Royal Infirmary of Edinburgh, Edinburgh, United Kingdom; c Usher Institute of Population Health Sciences and Informatics, University of Edinburgh, Edinburgh, United Kingdom; d Edinburgh Clinical Trials Unit, Usher Institute, University of Edinburgh, Edinburgh, United Kingdom; e Edinburgh Imaging Facility QMRI (The Queen’s Medical Research Institute), University of Edinburgh, Edinburgh, United Kingdom; f University of Glasgow, School of Medicine, Glasgow, United Kingdom

**Keywords:** acute coronary syndrome, coronary computed tomography angiogram, troponin, CAD, coronary artery disease, CCTA, coronary computed tomography angiography, CT, computed tomography, hs-cTn, high-sensitivity cardiac troponin

## Abstract

**Background:**

Patients with suspected acute coronary syndrome in whom myocardial infarction has been excluded are at risk of future adverse cardiac events.

**Objectives:**

This study evaluated the usefulness of high-sensitivity cardiac troponin I (hs-cTnI) to select patients for further investigation after myocardial infarction has been excluded.

**Methods:**

This is a prospective cohort study of patients presenting to the emergency department with suspected acute coronary syndrome and hs-cTnI concentrations below the sex-specific 99th percentile. Patients were recruited in a 2:1 fashion, stratified by peak hs-cTnI concentration above and below the risk stratification threshold of 5 ng/L. All patients underwent coronary computed tomography angiography (CCTA) after hospital discharge.

**Results:**

Overall, 250 patients were recruited (61.4 ± 12.2 years 31% women) in whom 62.4% (156 of 250 patients) had coronary artery disease (CAD). Patients with intermediate hs-cTnI concentrations (between 5 ng/L and the sex-specific 99th percentile) were more likely to have CAD than those with hs-cTnI concentrations <5 ng/L (71.9% [120 of 167 patients] vs 43.4% [36 of 83 patients]; odds ratio: 3.33; 95% CI: 1.92-5.78). Conversely, there was no association between anginal symptoms and CAD (63.2% [67 of 106 patients] vs 61.8% [89 of 144 patients]; odds ratio: 0.92; 95% CI: 0.48-1.76). Most patients with CAD did not have a previous diagnosis (53.2%; 83 of 156 patients) and were not on antiplatelet and statin therapies (63.5%; 99 of 156 patients) before they underwent CCTA.

**Conclusions:**

In patients who had myocardial infarction excluded, CAD was 3× more likely in those with intermediate hs-cTnI concentrations compared with low hs-cTnI concentrations. In such patients, CCTA could help to identify those with occult CAD and to target preventative treatments, thereby improving clinical outcomes.

Coronary artery disease (CAD) is the most common cause of death worldwide (1). In most patients, CAD remains undiagnosed until they present to hospital with acute chest pain (2). Current assessment strategies focus on ruling in or ruling out acute myocardial infarction through bedside clinical assessment, electrocardiography, and serial cardiac troponin testing (3). However, even after acute myocardial infarction has been ruled out, a significant proportion of patients may still have underlying CAD and are at risk of future adverse cardiac events. The optimal approach to select such patients for further investigation remains uncertain.

It is now increasingly recognized that high-sensitivity cardiac troponin (hs-cTn) concentrations within the normal reference range can aid in the triage of patients with suspected acute coronary syndrome. We previously identified and validated a rule-out threshold of 5 ng/L that maximizes the number of patients with suspected acute coronary syndrome who are identified as low risk at presentation with a negative predictive value of >99.5% for myocardial infarction or cardiac death at 30 days (4,5). This threshold has been incorporated into early rule-out pathways to expediate the evaluation of patients with suspected acute coronary syndrome (6). In contrast, those with intermediate troponin concentrations between 5 ng/L to 99th percentile diagnostic threshold are often triaged to further clinical observation and have substantially higher medium- and long-term risk of adverse cardiac events (4,5). We currently have limited understanding of the pathophysiological mechanisms for this observed increase in risk in those with intermediate troponin concentrations, and it is unclear whether this reflects unrecognized CAD. Insights here may help us develop evidence-based strategies to identify patients who are more likely to benefit from additional testing (7).

In patients with suspected acute coronary syndrome in whom myocardial infarction has been ruled out, we aim to determine whether those with intermediate troponin concentrations have a higher prevalence of CAD and whether troponin could be used to guide the selection of patients for coronary computed tomography angiography (CCTA).

## Methods

### Study design and population

PRECISE-CTCA (Troponin to Risk Stratify Patients with Acute Chest Pain for Computed Tomography Coronary Angiography) was a prospective cohort study (NCT04549805). Between December 4, 2018 and October 6, 2020, we prospectively enrolled 250 patients older than 30 years of age who presented to the emergency department at the Royal Infirmary of Edinburgh, United Kingdom, with suspected acute coronary syndrome in whom acute myocardial infarction had been ruled out and peak hs-cTn concentrations were within the normal reference range. Patients were recruited in a 2:1 fashion stratified by peak hs-cTnI (ARCHITECT_STAT_ troponin I assay; Abbott Laboratories) concentration above and below the risk stratification threshold of 5 ng/L (4,5).

Exclusion criteria were an inability to undergo CCTA due to severe renal failure (estimated glomerular filtration rate <30 mL/min/1.73 m^2^) or major allergy to iodinated contrast media, clear alternative diagnosis, requirement for in-patient investigation, CCTA, or invasive coronary angiogram within the past 1 year, pregnancy or breast feeding, and inability to give informed consent. This study was performed with approval of the South East Scotland Research Ethics Committee 01, and all participants provided written informed consent.

Presenting symptoms, cardiovascular risk factors, medical history, physiological measurements, clinical biochemistry and hematologic, and prescribed medications were recorded from participants at enrollment and from their electronic medical records. Symptoms of angina were defined using the Diamond and Forrester questions and classified as typical, atypical, or nonanginal chest pain as recommended by current national and international guidelines (8, 9, 10). Optimal preventative medical therapy was defined as a combination of antiplatelet and statin therapy.

### CCTA

All participants underwent CCTA as an outpatient procedure, as soon as possible after their initial hospital attendance, and the results were communicated to the patient and the responsible clinician with recommendations to commence secondary prevention if CAD was identified. CCTA was performed using a 128-detector row scanner (Biograph mCT, Siemens Healthcare) with iodine-based contrast media, as per Society of Cardiovascular Computed Tomography guidelines (11). Tube current and voltage were adjusted automatically on the basis of body habitus. Rate-limiting medication was administered for patients with a heart rate >60 beats/min. Sublingual glyceryl trinitrate was administered to all patients, unless contraindicated.

### Image analysis

CCTA images were reviewed by trained observers who performed a per-segment analysis using a 15-segment model to assess coronary artery stenoses, with complex cases classified by consensus. Luminal cross-sectional area stenoses were classified as normal (<10%), mild nonobstructive (10%-49%), moderate nonobstructive (50%-70%), or obstructive (>70% in ≥1 major epicardial artery or >50% in the left main stem). Patients were subsequently classified according to the most significant stenosis identified on the CCTA, regardless of whether the vessel has been stented. Coronary stenoses that were bypassed by a vascular graft were not considered in the classification. Atherosclerotic plaque burden was quantified using the segment involvement, segment stenosis, and computed tomography (CT)−adapted Leaman scores. The segment involvement score was calculated as the total number of segments with any plaque and ranged from 0 to 16 (12). The segment stenosis score also incorporated the severity of stenosis and ranged from 0 to 48. The CT-Leaman score incorporates weighting factors for the location of the plaque, the type of plaque (noncalcified, calcified, or mixed plaques) and the degree of stenosis, and ranges from 0 to 34.5 (13).

### Statistical analysis

Continuous variables were presented as mean ± SD or median (interquartile range) and compared using Student’s *t*-test or Mann-Whitney *U* test, whereas categorical variables were presented as n (%) and compared using chi-square or Fisher exact tests as appropriate. Prevalence of CAD in patients with intermediate troponin concentrations (5 ng/L to 99th percentile) was compared to those with low troponin concentrations (<5 ng/L) using logistic regression modeling adjusted for age, sex, smoking status, hypertension, diabetes mellitus, and estimated glomerular filtration rate. We also performed a post hoc sensitivity analysis restricted to patients without known CAD (defined as those without a previous diagnosis of angina and myocardial infarction). All analyses were performed in R version 4.0.3 (R Foundation for Statistical Computing).

### Sample size

In a previous study of patients who underwent CCTA for the investigation of possible angina, 46.8% of patients with intermediate troponin concentrations and 24.6% of patients with low troponin concentrations had obstructive CAD on CCTA (14). We estimated that a 2:1 recruitment of 250 patients above and below the 5 ng/L threshold would identify approximately 100 patients with obstructive CAD.

## Results

### Study population

We identified 759 patients with suspected acute coronary syndrome in whom myocardial infarction has been ruled out, of whom 250 patients were enrolled into our study (Supplemental Figure 1). Most patients who were not enrolled had ≥1 prespecified exclusion criteria following review of their history and electronic medical records by the research team. The study population consisted of 167 patients with intermediate troponin concentrations (between 5 ng/L and the sex-specific 99th percentile threshold: 16 ng/L for women and 34 ng/L for men) and 83 patients with low troponin concentrations (<5 ng/L). The mean age of study participants was 61 ± 12 years, and 31% (78 of 250) of participants were women (Table 1). Overall, 42.4% (106 of 250) of patients had anginal symptoms (12.8% [32 of 250 patients] had typical angina and 29.6% [74 of 250 patients] had atypical angina), with the remainder classified as having nonanginal chest pain (Table 1).

**Table 1:** Baseline Characteristics of Patients Stratified by Troponin Concentration

	Overall (N = 250)	<5 ng/L (n = 83)	5 ng/L to 99th Percentile (n = 167)	*P* Value
Men	172 (68.8)	51 (61.4)	121 (72.5)	0.104
Age, y	61.4 ± 12.2	56.8 ± 11.2	63.7 ± 12.0	<0.001
Presenting symptom				0.089
Chest pain	219 (87.6)	76 (91.6)	143 (85.6)	
Dyspnea	5 (2.0)	0 (0.0)	5 (3.0)	
Palpitations	19 (7.6)	3 (3.6)	16 (9.6)	
Other	7 (2.8)	4 (4.8)	3 (1.8)	
Anginal symptoms	106 (42.4)	30 (36.1)	76 (45.5)	0.202
Typical angina	32 (12.8)	6 (7.2)	26 (15.6)	0.097
Atypical angina	74 (29.6)	24 (28.9)	50 (29.9)	0.984
Cardiovascular risk factors				
BMI, kg/m^2^	29.5 ± 6.0	29.8 ± 6.4	29.3 ± 5.8	0.579
Current or previous cigarette smoker	136 (54.4)	47 (56.6)	89 (53.3)	0.716
Hypertension	109 (43.6)	30 (36.1)	79 (47.3)	0.123
Diabetes mellitus	35 (14.0)	5 (6.0)	30 (18.0)	0.018
Hyperlipidemia	53 (21.3)	20 (24.1)	33 (19.9)	0.547
Family history of CAD	92 (36.8)	28 (33.7)	64 (38.3)	0.569
Medical history				
Angina	45 (18.0)	11 (13.3)	34 (20.4)	0.229
Myocardial infarction	52 (20.8)	12 (14.5)	40 (24.0)	0.115
Stroke	15 (6.0)	2 (2.4)	13 (7.8)	0.161
Peripheral vascular disease	8 (3.2)	2 (2.4)	6 (3.6)	0.905
Atrial fibrillation	19 (7.6)	4 (4.8)	15 (9.0)	0.36
Chronic kidney disease	22 (8.8)	3 (3.6)	19 (11.4)	0.071
Previous revascularization				
PCI	53 (21.2)	13 (15.7)	40 (24.0)	0.178
CABG	12 (4.8)	2 (2.4)	10 (6.0)	0.351
Medications at presentation				
Aspirin	61 (24.4)	14 (16.9)	47 (28.1)	0.072
P2Y_12_ inhibitor	19 (7.6)	4 (4.8)	15 (9.0)	0.36
Statin	105 (42.0)	24 (28.9)	81 (48.5)	0.005
ACEi or ARB	91 (36.4)	24 (28.9)	67 (40.1)	0.111
Beta-blocker	69 (27.6)	21 (25.3)	48 (28.7)	0.672
Oral anticoagulant	26 (10.4)	5 (6.0)	21 (12.6)	0.168
Physiology and investigations				
Myocardial ischemia on ECG	9 (3.6)	1 (1.2)	8 (4.8)	0.283
Heart rate, beats/min	76 ± 18	77 ± 17	76 ± 18	0.89
Systolic blood pressure, mm Hg	150 ± 26	149 ± 24	151 ± 27	0.58
Hemoglobin, g/L	144 ± 14	146 ± 13	143 ± 15	0.197
eGFR, mL/min/1.73 m^2^	84 ± 17	89 ± 13	82 ± 18	0.001
Total cholesterol, mmol/L	4.9 ± 1.2	5.0 ± 1.1	4.8 ± 1.2	0.151
LDL cholesterol, mmol/L	3.0 ± 1.1	3.2 ± 1.0	3.0 ± 1.2	0.106
Peak troponin I concentration, ng/L	6.0 (3.0-10.0)	2.0 (1.0-3.0)	8.0 (6.0-12.0)	<0.001
TIMI risk score	1.6 (1.3)	1.2 (1.1)	1.8 (1.3)	<0.001
GRACE risk score	92.5 (25.2)	86.8 (24.6)	96.4 (25.0)	0.008
Time intervals				
Symptom onset to first troponin test, h	9 (3-31)	8 (3-24)	9 (4-45)	0.099
First to second troponin test, h	3 (2-3)	3 (3-3)	3 (2-3)	0.737
Presentation to outpatient CCTA, d	22 (15-30)	17 (8-25)	24 (19-31)	<0.001

Values are n (%), mean ± SD, or median (interquartile range).

ACEi = angiotensin-converting enzyme inhibitor; ARB = angiotensin receptor blocker; BMI = body mass index; CABG = coronary artery bypass grafting; CAD = coronary artery disease; CCTA = coronary computed tomography angiography; ECG = electrocardiogram; eGFR = estimated glomerular filtration rate; LDL = low-density lipoprotein; PCI = percutaneous coronary intervention; TIMI = Thrombolysis In Myocardial Infarction.

Patients with intermediate troponin concentrations were older than those with low troponin concentrations (64 ± 12 years vs 57 ± 11 years, respectively; *P* < 0.001) and were more likely to have diabetes mellitus (18% [30 of 167 patients] vs 6% [5 of 83 patients]; *P* = 0.018). Otherwise, both groups had a similar proportion of women, presenting symptoms, cardiovascular risk factors, medical history, and history of coronary revascularization. At presentation, patients with intermediate troponin concentrations had higher Thrombolysis In Myocardial Infarction (15) and Global Registry of Acute Coronary Events (16) risk scores (1.8 ± 1.3 vs 1.2 ± 1.1; *P* < 0.001 and 96.4 ± 25.0 vs 86.8 ± 24.6; *P* = 0.008, respectively) and were more likely to be on antiplatelet (28.1% [47 of 167 patients] vs 16.9% [14 of 83 patients]; *P* = 0.072) and statin (48.5% [81 of 167 patients] vs 28.9% [24 of 83 patients]; *P* = 0.005) therapies than those with low troponin concentrations.

### CCTA

Overall, 37.6% (94 of 250) of patients had normal coronary arteries on CCTA, 36.0% (90 of 250 patients) had nonobstructive disease, and 26.4% (66 of 250 patients) had obstructive disease (Table 2).

**Table 2:** Findings on CCTA Stratified by Troponin Concentration

	Overall (N = 250)	<5 ng/L (n = 83)	5 ng/L to 99th Percentile (n = 167)	*P* Value
Stenosis severity				<0.001
Normal	94 (37.6)	47 (56.6)	47 (28.1)	
Nonobstructive CAD	90 (36.0)	20 (24.1)	70 (41.9)	
Mild (<50%)	65 (26.0)	13 (15.7)	52 (31.1)	
Moderate (50-70%)	25 (10.0)	7 (8.4)	18 (10.8)	
Obstructive CAD	66 (26.4)	16 (19.3)	50 (29.9)	
1 Vessel	33 (13.2)	6 (7.2)	27 (16.2)	
2 Vessels	22 (8.8)	8 (9.6)	14 (8.4)	
3 Vessels	11 (4.4)	2 (2.4)	9 (5.4)	
Atherosclerotic burden				
Segment involvement score	2.0 (0.0-6.0)	0.0 (0.0-3.0)	2.0 (0.0-6.0)	<0.001
Segment stenosis score	2.0 (0.0-8.0)	0.0 (0.0-4.0)	3.0 (0.0-9.0)	<0.001
CT-Leaman score	3.2 (0.0-9.8)	0.0 (0.0-6.2)	5.2 (0.0-10.4)	<0.001

Values are n (%), or median (interquartile range).

Abbreviations as in Table 1.

**Figure 1:**
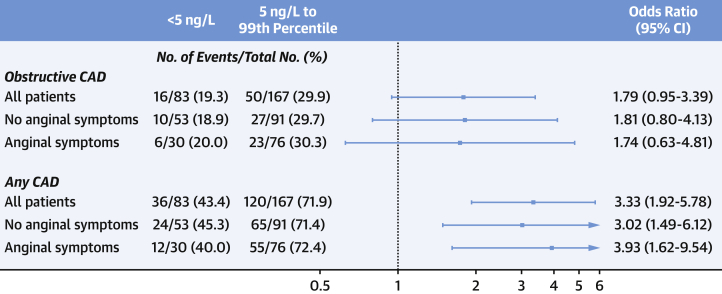
Association Between High-Sensitivity Cardiac Troponin and CAD Odds ratio of any coronary artery disease (CAD) and obstructive CAD on coronary computed tomography angiography in all patients with intermediate (between 5 ng/L and the sex-specific 99th percentile) versus low troponin concentrations (<5 ng/L) and stratified by the presence of anginal symptoms. CI = confidence interval.

Patients with intermediate troponin concentrations were more likely to have CAD than those with low troponin concentrations (71.9% [120 of 167 patients] vs 43.4% [36 of 83 patients]; odds ratio: 3.33; 95% CI: 1.92-5.78 for any CAD, and 29.9% [50 of 167 patients] vs 19.3% [16 of 83 patients]; odds ratio: 1.79; 95% CI: 0.95-3.39 for obstructive disease) (Figure 1). They also had more atherosclerotic plaque burden (median segment involvement score of 2.0 [0.0-6.0] vs 0.0 [0.0-3.0]; segment stenosis score of 3.0 [0.0-9.0] vs 0.0 [0.0-4.0]; and CT-Leaman score 5.2 [0.0-10.4] vs 0.0 [0.0-6.2]; *P* < 0.001 for all). These associations persisted in multivariate analysis (Supplemental Figure 2). Similar findings were observed in a sensitivity analysis restricted to patients without known CAD (Supplemental Tables 1 and 2, and Supplemental Figure 3).

**Figure 2:**
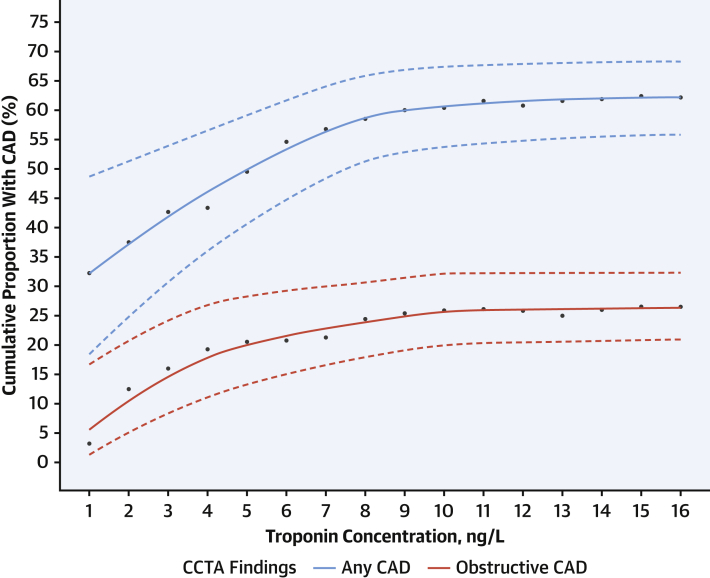
Cumulative Proportion With CAD Across Troponin Concentrations Cumulative proportion of patients with CAD across troponin concentrations within the normal reference range. **Solid lines** represent central estimate and **dashed lines** represent 95% CIs. CCTA = coronary computed tomography angiography; other abbreviation as Figure 1.

Patients with obstructive and nonobstructive CAD had higher median troponin concentrations (7.5 ng/L [interquartile range: 5.0-10.0 ng/L] and 7.0 ng/L [interquartile range: 5.0-10.0 ng/L], respectively) compared with those with normal coronary arteries (4.5 ng/L [interquartile range: 2.0-8.0 ng/L]; *P* = 0.001 for both) (Supplemental Table 3). As troponin concentration increased within the normal reference range, the cumulative proportion of patients identified with any CAD increased from 32.3% (10 of 31) in patients with troponin concentrations below the limit of detection of 1.2 ng/L to 62.2% (143 of 230) in those with troponin concentrations of ≤16 ng/L (Figure 2). Across this range of troponin concentrations, the cumulative proportion with obstructive CAD increased from 3.2% (1 of 31 patients) to 26.5% (61 of 230 patients).

### Anginal symptoms

There were no differences in the prevalence of CAD between those with and without anginal symptoms (63.2% [67 of 106 patients] vs 61.8% [89 of 144 patients]; odds ratio: 1.06; 95% CI: 0.63-1.78 for any CAD, and 27.4% [29 of 106 patients] vs 25.7% [37 of 144 patients]; odds ratio: 1.09; 95% CI: 0.62-1.92 for obstructive disease) (Supplemental Table 4, Supplemental Figure 4). Atherosclerotic plaque burden was also similar in those with and without symptoms of angina (median segment involvement score of 2.0 [0.0-6.0] vs 2.0 [0.0-5.2]; *P* = 0.787, segment stenosis score of 2.0 [0.0-8.0] vs 2.0 [0.0-7.2]; *P* = 0.692 and CT-Leaman score 3.2 [0.0-10.3] vs 3.2 [0.0-9.4]; *P* = 0.749). Patients with intermediate troponin concentrations had a higher prevalence of CAD compared with those with low troponin concentrations in both those with (72.4% [55 of 76 patients] vs 40.0% [12 of 30 patients]; odds ratio: 3.93; 95% CI: 1.62-9.54) and without anginal symptoms (71.4% [65 of 91 patients] vs 45.3% [24 of 53 patients]; odds ratio: 3.02; 95% CI: 1.49-6.12) (Figure 1).

### Diagnosis and treatment of CAD

**Figure 3:**
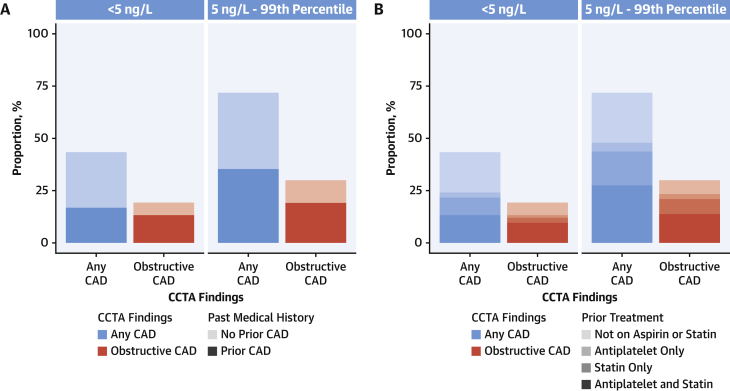
Previous Diagnosis and Treatment in Patients With CAD Identified on CCTA **(A)** Proportion of patients with CAD identified on CCTA with low (<5 ng/L) and intermediate troponin concentrations (between 5 ng/L and the sex-specific 99th percentile) stratified by medical history of CAD. **(B)** Proportion of patients with CAD identified on CCTA with low (<5 ng/L) and intermediate troponin concentrations (between 5 ng/L and the sex-specific 99th percentile) stratified by previous treatment with preventative medical therapy. Abbreviations as in Figures 1 and 2.

Most patients with CAD identified on CCTA did not have a history of CAD (50.8% [61 of 120] and 61.0% [22 of 36] in patients with intermediate and low troponin concentrations, respectively) (Figure 3A). Most were not on optimal preventative medical therapy for CAD before undergoing CCTA (61.7% [74 of 120] and 69.4% [25 of 36] in patients with intermediate and low troponin concentrations, respectively) (Figure 3B).

## Discussion

In this study, we evaluated the use of CCTA in patients who presented to the hospital with suspected acute coronary syndrome after myocardial infarction had been ruled out. We found that despite patients having cardiac troponin concentrations within the normal reference range, CCTA identified CAD in two-thirds of patients with intermediate cardiac troponin concentrations. Although a troponin concentration below the rule-out threshold of 5 ng/L did not exclude CAD, patients with intermediate troponin concentrations were 3× more likely to have CAD and had a greater atherosclerotic plaque burden. These associations persisted after adjusting for age, sex, and cardiovascular risk factors, and was present, regardless of whether patients had symptoms of angina. Conversely, the prevalence and burden of CAD were similar in those with and without symptoms of angina. Furthermore, most patients with CAD identified by CCTA did not have a previous diagnosis and were not prescribed optimal preventative medical therapy before undergoing CCTA.

We believe our findings extended the role of hs-cTn in the assessment of patients with suspected acute coronary syndrome (Central Illustration). At present, hs-cTn is primarily applied to rule in and rule out acute myocardial infarction using fixed thresholds (3). However, it is now increasingly recognized that cardiac troponin concentrations within the normal reference range are a continuous marker of risk and can be used to improve risk stratification further (17, 18, 19). The discovery that low troponin concentrations identify patients who are at low risk of cardiac events has led to the development of accelerated care pathways for patients with suspected acute coronary syndrome (6,20, 21, 22). Recent evidence from randomized controlled trials has shown that implementation of this approach is safe and effective at substantially reducing length of hospital stay and the proportion of patients who require hospital admission (23). In contrast, among patients in whom myocardial infarction has been ruled out, an intermediate troponin concentration within the normal reference range is associated with a 5-10× higher medium- and long-term risk of adverse cardiac events compared with those with a low (<5 ng/L) troponin concentration (4,5). The optimal approach to the investigation and management of these patients is unknown. Recent data from the RAPID-TnT (Rapid Assessment of Possible Acute Coronary Syndrome in the Emergency Department with High-Sensitivity Troponin T) randomized trial demonstrated that implementation of hs-cTnT and a 0/1-h pathway was associated with an increase in subsequent myocardial infarction and death at 1 year in patients with intermediate cardiac troponin concentrations compared with that of standard care (24). The explanation for these unexpected findings was not clear, but the investigators reported lower use of functional testing and follow-up in the intervention arm. Our finding that many of these patients had underlying unrecognized underlying CAD and were not on preventative medical therapy before undergoing CCTA suggested that targeting this at-risk group could present an opportunity to improve outcomes.

**Central Illustration:**
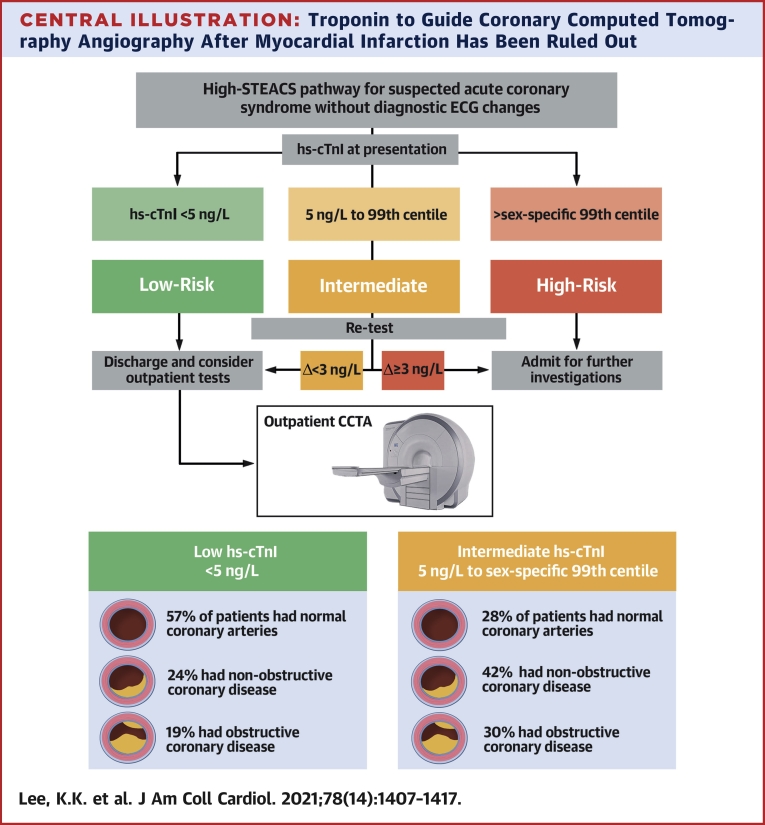
Troponin to Guide Coronary Computed Tomography Angiography After Myocardial Infarction Has Been Ruled Out In this prospective cohort study, 250 patients with suspected acute coronary syndrome underwent outpatient coronary computed tomography angiography (CCTA) after acute myocardial infarction was ruled out. Patients with intermediate high-sensitivity cardiac troponin I concentrations (between 5 ng/L and the sex-specific 99th percentile) were 3× more likely to have coronary artery disease (CAD) than those with high-sensitivity cardiac troponin I concentrations <5 ng/L. This approach to use cardiac troponin to select patients for downstream CCTA after myocardial infarction has been ruled out has the major potential to improve patient outcomes by improving the diagnosis of CAD and use of preventative treatments. High-STEACS = High-Sensitivity Troponin in the Evaluation of patients with suspected Acute Coronary Syndrome; ECG = electrocardiogram.

There were several potential underlying mechanisms through which hs-cTn concentrations could inform the future risk of adverse cardiac events. Although cardiac troponin could not exclude CAD because 4 in 10 patients with troponin concentrations <5 ng/L in our study had some evidence of disease, troponin reflected the atherosclerotic plaque burden and downstream consequences of disease. Patients with CAD who have plaque instability are more likely to have subclinical myocardial necrosis, which may explain the increased risk in those with intermediate troponin concentrations (25, 26, 27, 28, 29). The prevalence of any CAD in patients with troponin concentrations of <5 ng/L (43.1%) was identical to the prevalence in 25,181 middle-age persons randomly selected for CCTA from the general population (42.0%) in the SCAPIS (Swedish Cardiopulmonary Bioimage Study) (30). This observation provided indirect support for the use of hs-cTn to target CCTA, following the exclusion of myocardial infarction to identify patients who were more likely to have CAD than the general population and were at increased risk of future cardiovascular events.

Current international guidelines recommend the use of clinical judgment to select patients for invasive and noninvasive imaging after myocardial infarction has been ruled out (3). In routine clinical practice, this primarily involves the careful evaluation of the patient’s history for symptoms of angina (8). In our study, we found that the prevalence of CAD was similar in those with and without symptoms of typical or atypical angina. Conversely, in patients with and without symptoms of angina, intermediate hs-cTn concentrations identified those with a significantly higher prevalence of CAD. This finding was consistent with previous studies in patients with stable chest pain (31) and raised the question as to whether an alternative approach that utilizes an objective test (eg, cardiac troponin) might be more accurate in selecting patients with a higher pretest probability of CAD for further diagnostic testing, regardless of symptoms.

There is now increasing evidence to support the use of CCTA over functional ischemia testing in patients with stable chest pain (32,33). Recent trials demonstrated that CCTA clarified the diagnosis of CAD and led to major improvements in patient outcomes by increasing the use of evidence-based preventative therapies (34). Furthermore, CCTA was cost-effective and led to less use of further downstream testing compared with functional testing (35,36). Secondary analyses of multiple randomized controlled trial data also showed that anatomical information provided by CCTA was an excellent predictor of prognosis (37, 38, 39, 40). In contrast, the extent of ischemia on functional testing was a poor discriminator of future risk (41). These data suggest that CCTA is likely to provide better diagnostic and prognostic information than that of functional testing to guide patient care once myocardial infarction has been ruled out.

In our current practice, the role of CCTA in patients with suspected acute coronary syndrome was not well defined. Previous studies primarily focused on the use of CCTA to aid in the diagnosis of acute myocardial infarction (42, 43, 44, 45, 46). Although several studies showed that CCTA could improve the accuracy and efficiency of ruling out myocardial infarction when used in addition to previous generations of troponin assays, CCTA was no longer able to improve patient flow in comparison with a standard of care that utilized hs-cTn (47). In contrast to these previous studies, we proposed an approach that combined hs-cTn and CCTA after acute myocardial infarction had been ruled out. We believe our findings built on evidence from previous studies that demonstrated that CCTA improved the diagnosis of CAD across a wide spectrum of pretest probability (48,49). Although CCTA will likely have a greater impact on care in those without known disease, only two-thirds of patients with known CAD in our study were on optimal medical therapy (67%; 55 of 82 patients). Furthermore, these patients presented with acute chest pain, and many had symptoms consistent with typical or atypical angina. CCTA can be useful in both patients with and without known CAD to determine the nature of their symptoms, optimize use of secondary prevention, and to guide revascularization in those with persistent symptoms despite optimal medical therapy or those with prognostically important CAD, such as severe 3-vessel or left main stem stenosis.

### Study limitations

We evaluated the severity of CAD based on the degree and extent of coronary artery stenosis rather than performing more detailed analysis of plaque phenotype to identify high-risk plaque features or an evaluation of coronary physiology using CT fractional flow reserve. Although we used coronary artery stenosis to reflect current clinical practice, we acknowledge that plaque phenotype and CT fractional flow reserve are promising advances in the evaluation of CAD on CCTA and merit further investigation (38,50,51). We also acknowledge that the prevalence of obstructive CAD in our cohort was lower than anticipated, and this reduced the power of our analysis. All participants in this study individually consented to the study. Although our researchers were embedded within the usual clinical care team in the emergency department and endeavored to enroll all potentially eligible patients using our prespecified eligibility criteria, it was possible that this approach introduced the selection bias that is inherent to studies that rely on individual patient consent. Nevertheless, we achieved a high recruitment rate with one-third of all potentially eligible patients consenting to participate in our study. Finally, this was an observational study, and therefore, we were not able to directly evaluate the impact of selecting patients with intermediate cardiac troponin for CCTA on outcomes. The TARGET-CTCA (Troponin in Acute Chest Pain to Risk Stratify and Guide Effective Use of Computed Tomography Coronary Angiography) study is currently evaluating this in a multicenter randomized controlled trial (52).

## Conclusions

In patients who had myocardial infarction ruled out, those with intermediate troponin concentrations were 3× more likely to have CAD on CCTA than those with low troponin concentrations. Conversely, the presence of anginal symptoms did not discriminate between those with and without CAD. Most patients with CAD did not have a previous diagnosis and were not on optimal preventative medical therapy. This approach to use cardiac troponin to select patients for downstream investigation after myocardial infarction has been ruled out has major potential to improve patient outcomes.

Perspectives **COMPETENCY IN PATIENT CARE AND PROCEDURAL SKILLS:** In patients with suspected acute coronary syndrome without myocardial infarction, intermediate blood levels of hs-cTn identify those with a higher prevalence of CAD on CCTA compared with those with low troponin levels.

**TRANSLATIONAL OUTLOOK:** Clinical trials are needed to assess the impact on clinical outcomes of guiding selection of patients for coronary interventions based on cardiac troponin levels.

## Funding Support and Author Disclosures

Dr Lee was supported by a British Heart Foundation (BHF) Clinical Research Training Fellowship (FS/18/25/33454). Drs Bularga and Wereski and Mr. Doudesis were supported by the Medical Research Council (MR/N013166/1, MR/V007017/1, MR/V007254/1). Dr Williams was supported by the British Heart Foundation (FS/ICRF/20/26002). Dr van Beek was supported by the Scottish Imaging Network. Dr Mills was supported by a Chair Award (CH/F/21/90010), Programme Grant (RG/20/10/34966), and Research Excellence Award (RE/18/5/34216) from the British Heart Foundation. Dr Lee has received honoraria from Abbott Diagnostics. Dr van Beek is founder/owner of QCTIS Ltd; has received honoraria from Aidence NV, Roche Diagnostics, AstraZeneca, and Mentholatum; and has received research support from Siemens Healthineers. Dr Mills has received honoraria from Abbott Diagnostics, Siemens Healthineers, Roche Diagnostics, and LumiraDx; and the University of Edinburgh has received research grants from Abbott Diagnostics and Siemens Healthineers. All other authors have reported that they have no relationships relevant to the contents of this paper to disclose.
