# Supplementary material for: Troponin-Guided Coronary Computed Tomographic Angiography After Exclusion of Myocardial Infarction
Source: J Am Coll Cardiol. 2021 Oct 5;78(14):1407–17. doi: 10.1016/j.jacc.2021.07.055 (PMC8482793; doi:10.1016/j.jacc.2021.07.055)
Supplement: Supplemental Tables 1–4 and Supplemental Figures 1–4 [file mmc1.docx]

##### Supplemental Material

**Troponin-Guided Coronary Computed Tomography Angiography After Exclusion of Myocardial Infarction**

Kuan Ken Lee, MD,^1^ Anda Bularga, MD,^1^ Rachel O’Brien, BN,^2^ Amy V. Ferry, PhD,^1^

Dimitrios Doudesis, MSc,^1, 3^ Takeshi Fujisawa, PhD,^1^ Shauna Kelly, BSc,^1^

Stacey Stewart, BSc, ^1^ Ryan Wereski, MD,^1^ Denise Cranley, BSc,^4^

Edwin J. R. van Beek, MD,^1,6^ David J Lowe, MD,^5^ David E Newby, MD,^1^

Michelle C Williams, MBChB,^1,6^ Alasdair J Gray, MD,^2, 3^ Nicholas L Mills, MD^1, 3^

^1^ BHF Centre for Cardiovascular Science, University of Edinburgh, Edinburgh, UK.

^2^ Department of Emergency Medicine, Emergency Medicine Research Group, Royal Infirmary of Edinburgh, Edinburgh, UK.

^3^ Usher Institute of Population Health Sciences and Informatics, University of Edinburgh, Edinburgh, UK.

^4^ Edinburgh Clinical Trials Unit, Usher Institute, University of Edinburgh, Edinburgh, UK.

^5^ University of Glasgow, School of Medicine, Glasgow, UK.

^6^ Edinburgh Imaging facility QMRI, University of Edinburgh, Edinburgh, UK.

**Corresponding Author:**

Professor Nicholas L Mills

BHF/University Centre for Cardiovascular Science

The University of Edinburgh

Edinburgh EH16 4SA

United Kingdom

Telephone: 0044 131 242 6515

E-mail: [nick.mills@ed.ac.uk](mailto:nick.mills@ed.ac.uk)

**Supplemental Tables:** 4

**Supplemental Figures:** 4

**Supplemental Table 1.** Baseline characteristics of patients without known coronary artery disease stratified by troponin concentration.

|  | **Overall** | **<5ng/L** | **5ng/L to**  **99^th^ centile** | P-value |
| --- | --- | --- | --- | --- |
| **Number of participants** | 168 | 65 | 103 |  |
| **Men** | 108 (64.3) | 40 (61.5) | 68 (66.0) | 0.671 |
| **Age, years** | 58.5 (12.2) | 54.4 (10.3) | 61.2 (12.6) | <0.001 |
| **Presenting symptom** |  |  |  | 0.013 |
| Chest pain | 146 (86.9) | 62 (95.4) | 84 (81.6) |  |
| Dyspnoea | 3 (1.8) | 0 (0.0) | 3 (2.9) |  |
| Palpitations | 16 (9.5) | 1 (1.5) | 15 (14.6) |  |
| Other | 3 (1.8) | 2 (3.1) | 1 (1.0) |  |
| **Anginal symptoms** | 62 (36.9) | 20 (30.8) | 42 (40.8) | 0.252 |
| Typical angina | 20 (11.9) | 4 (6.2) | 16 (15.5) | 0.113 |
| Atypical angina | 42 (25.0) | 16 (24.6) | 26 (25.2) | 1 |
| **Cardiovascular risk factors** |  |  |  |  |
| BMI | 29.7 (6.2) | 29.9 (6.8) | 29.5 (5.9) | 0.667 |
| Current or previous cigarette smoker | 86 (51.2) | 34 (52.3) | 52 (50.5) | 0.943 |
| Hypertension | 63 (37.5) | 22 (33.8) | 41 (39.8) | 0.54 |
| Diabetes | 15 (8.9) | 3 (4.6) | 12 (11.7) | 0.201 |
| Hyperlipidemia | 46 (27.5) | 19 (29.2) | 27 (26.5) | 0.832 |
| Family history of CAD | 56 (33.3) | 23 (35.4) | 33 (32.0) | 0.779 |
| **Past medical history** |  |  |  |  |
| Stroke | 6 (3.6) | 1 (1.5) | 5 (4.9) | 0.483 |
| Peripheral vascular disease | 2 (1.2) | 1 (1.5) | 1 (1.0) | 1 |
| Atrial fibrillation | 9 (5.4) | 2 (3.1) | 7 (6.8) | 0.49 |
| **Medications at presentation** |  |  |  |  |
| Aspirin | 9 (5.4) | 2 (3.1) | 7 (6.8) | 0.49 |
| P2Y12 inhibitor | 5 (3.0) | 1 (1.5) | 4 (3.9) | 0.685 |
| Statin | 39 (23.2) | 9 (13.8) | 30 (29.1) | 0.036 |
| ACEi or ARB | 40 (23.8) | 13 (20.0) | 27 (26.2) | 0.462 |
| Beta-blocker | 29 (17.3) | 12 (18.5) | 17 (16.5) | 0.907 |
| Oral anticoagulant | 12 (7.1) | 2 (3.1) | 10 (9.7) | 0.187 |
| **Physiology and investigations** |  |  |  |  |
| Myocardial ischaemia on ECG | 6 (3.6) | 0 (0.0) | 6 (5.8) | 0.12 |
| Heart rate, beats per minute | 80 (17) | 79 (17) | 80 (18) | 0.776 |
| Systolic blood pressure, mmHg | 152 (26) | 150 (25) | 153 (26) | 0.541 |
| Haemoglobin, g/L | 144 (14) | 146 (14) | 143 (14) | 0.224 |
| eGFR, mL/min/1.73 m^2^ | 88 (16) | 92 (12) | 85 (17) | 0.012 |
| Total cholesterol, mmol/L | 5.2 (1.1) | 5.3 (1.0) | 5.2 (1.1) | 0.475 |
| LDL cholesterol, mmol/L | 3.3 (1.0) | 3.4 (0.9) | 3.2 (1.0) | 0.234 |
| Peak troponin I concentration, ng/L | 6.0 [2.0, 9.2] | 2.0 [1.0, 3.0] | 8.0 [6.0, 12.0] | <0.001 |
| TIMI risk score | 0.9 (0.8) | 0.8 (0.7) | 1.0 (0.8) | 0.037 |
| GRACE risk score | 86.3 (24.6) | 81.3 (20.0) | 90.7 (27.5) | 0.026 |

Presented as No. (%), mean (SD) or median [inter-quartile range].

Abbreviations: BMI= body mass index; CAD= coronary artery disease; PCI= percutaneous coronary intervention; CABG= coronary artery bypass grafting; ACEi= angiotensin converting enzyme inhibitor; ARB= angiotensin receptor blocker; ECG= electrocardiogram; eGFR= estimated glomerular filtration rate.

**Supplemental Table 2.** Findings on coronary computed tomography angiography in patients without known coronary artery disease stratified by troponin concentration.

|  | **Overall** | **<5ng/L** | **5ng/L to**  **99^th^ centile** | P-value |
| --- | --- | --- | --- | --- |
| **Number of participants** | 168 | 65 | 103 |  |
| **Stenosis severity** |  |  |  | 0.01 |
| Normal | 85 (50.6) | 43 (66.2) | 42 (40.8) |  |
| Non-obstructive CAD | 60 (35.7) | 17 (26.2) | 43 (41.7) |  |
| Mild (<50%) | 44 (26.2) | 11 (16.9) | 33 (32.0) |  |
| Moderate (50-70%) | 16 (9.5) | 6 (9.2) | 10 (9.7) |  |
| Obstructive CAD | 23 (13.7) | 5 (7.7) | 18 (17.5) |  |
| One vessel | 16 (9.5) | 4 (6.2) | 12 (11.7) |  |
| Two vessels | 3 (1.8) | 0 (0.0) | 3 (2.9) |  |
| Three vessels | 4 (2.4) | 1 (1.5) | 3 (2.9) |  |
| **Atherosclerotic burden** |  |  |  |  |
| Segment involvement score | 0.0 [0.0, 3.0] | 0.0 [0.0, 1.0] | 1.0 [0.0, 4.0] | 0.001 |
| Segment stenosis score | 0.0 [0.0, 4.0] | 0.0 [0.0, 1.0] | 2.0 [0.0, 5.0] | 0.001 |
| CT Leaman score | 0.0 [0.0, 5.5] | 0.0 [0.0, 2.4] | 2.8 [0.0, 7.6] | 0.001 |

Presented as No. (%), mean (SD) or median [inter-quartile range].

Abbreviations: CAD= coronary artery disease; CCTA=coronary computed tomography angiography.

**Supplemental Table 3.** Baseline characteristics of patients stratified by findings on coronary computed tomography angiography.

|  | **Overall** | **Normal** | **Non-obstructive CAD** | **Obstructive CAD** | P-value |
| --- | --- | --- | --- | --- | --- |
| **Number of participants** | 250 | 94 | 90 | 66 |  |
| **Men** | 172 (68.8) | 55 (58.5) | 65 (72.2) | 52 (78.8) | 0.017 |
| **Age, years** | 61.4 (12.2) | 53.9 (11.4) | 63.5 (10.4) | 69.4 (9.0) | <0.001 |
| **Presenting symptom** |  |  |  |  | 0.86 |
| Chest pain | 219 (87.6) | 83 (88.3) | 77 (85.6) | 59 (89.4) |  |
| Dyspnoea | 5 (2.0) | 1 (1.1) | 2 (2.2) | 2 (3.0) |  |
| Palpitations | 19 (7.6) | 8 (8.5) | 7 (7.8) | 4 (6.1) |  |
| Other | 7 (2.8) | 2 (2.1) | 4 (4.4) | 1 (1.5) |  |
| **Anginal symptoms** | 106 (42.4) | 39 (41.5) | 38 (42.2) | 29 (43.9) | 0.953 |
| Typical angina | 32 (12.8) | 10 (10.6) | 11 (12.2) | 11 (16.7) | 0.521 |
| Atypical angina | 74 (29.6) | 29 (30.9) | 27 (30.0) | 18 (27.3) | 0.883 |
| **Cardiovascular risk factors** |  |  |  |  |  |
| BMI | 29.5 (6.0) | 29.0 (4.7) | 30.6 (7.2) | 28.6 (5.5) | 0.077 |
| Current or previous cigarette smoker | 136 (54.4) | 43 (45.7) | 49 (54.4) | 44 (66.7) | 0.033 |
| Hypertension | 109 (43.6) | 24 (25.5) | 47 (52.2) | 38 (57.6) | <0.001 |
| Diabetes | 35 (14.0) | 4 (4.3) | 16 (17.8) | 15 (22.7) | 0.002 |
| Hyperlipidemia | 53 (21.3) | 22 (23.7) | 23 (25.6) | 8 (12.1) | 0.1 |
| Family history of CAD | 92 (36.8) | 33 (35.1) | 37 (41.1) | 22 (33.3) | 0.555 |
| **Past medical history** |  |  |  |  |  |
| Angina | 45 (18.0) | 6 (6.4) | 15 (16.7) | 24 (36.4) | <0.001 |
| Myocardial infarction | 52 (20.8) | 5 (5.3) | 18 (20.0) | 29 (43.9) | <0.001 |
| Stroke | 15 (6.0) | 2 (2.1) | 6 (6.7) | 7 (10.6) | 0.08 |
| Peripheral vascular disease | 8 (3.2) | 0 (0.0) | 2 (2.2) | 6 (9.1) | 0.005 |
| Atrial fibrillation | 19 (7.6) | 1 (1.1) | 12 (13.3) | 6 (9.1) | 0.006 |
| **Previous revascularisation** |  |  |  |  |  |
| PCI | 53 (21.2) | 4 (4.3) | 17 (18.9) | 32 (48.5) | <0.001 |
| CABG | 12 (4.8) | 0 (0.0) | 8 (8.9) | 4 (6.1) | 0.016 |
| **Medications at presentation** |  |  |  |  |  |
| Aspirin | 61 (24.4) | 8 (8.5) | 23 (25.6) | 30 (45.5) | <0.001 |
| P2Y12 inhibitor | 19 (7.6) | 3 (3.2) | 8 (8.9) | 8 (12.1) | 0.094 |
| Statin | 105 (42.0) | 14 (14.9) | 46 (51.1) | 45 (68.2) | <0.001 |
| ACEi or ARB | 91 (36.4) | 17 (18.1) | 35 (38.9) | 39 (59.1) | <0.001 |
| Beta-blocker | 69 (27.6) | 19 (20.2) | 22 (24.4) | 28 (42.4) | 0.006 |
| Oral anticoagulant | 26 (10.4) | 5 (5.3) | 11 (12.2) | 10 (15.2) | 0.104 |
| **Physiology and investigations** |  |  |  |  |  |
| Myocardial ischaemia on ECG | 9 (3.6) | 3 (3.2) | 5 (5.6) | 1 (1.5) | 0.394 |
| Heart rate, beats per minute | 76 (18) | 77 (19) | 79 (17) | 73 (16) | 0.18 |
| Systolic blood pressure, mmHg | 150 (26) | 152 (24) | 149 (28) | 149 (25) | 0.738 |
| Haemoglobin, g/L | 144 (14) | 144 (15) | 145 (14) | 144 (13) | 0.871 |
| eGFR, mL/min/1.73 m^2^ | 84 (17) | 89 (15) | 84 (17) | 78 (16) | <0.001 |
| Total cholesterol, mmol/L | 4.9 (1.2) | 5.2 (1.0) | 4.9 (1.2) | 4.5 (1.1) | 0.002 |
| LDL cholesterol, mmol/L | 3.0 (1.1) | 3.3 (0.9) | 3.1 (1.2) | 2.7 (1.3) | 0.003 |
| Peak troponin I concentration, ng/L | 6.0 [3.0, 10.0] | 4.5 [2.0, 8.0] | 7.0 [5.0, 10.0] | 7.5 [5.0, 10.0] | 0.001 |
| TIMI risk score | 1.6 (1.3) | 1.0 (1.0) | 1.6 (1.2) | 2.5 (1.2) | <0.001 |
| GRACE risk score | 92.5 (25.2) | 76.5 (20.2) | 98.7 (22.2) | 108.2 (22.3) | <0.001 |

Presented as No. (%), mean (SD) or median [inter-quartile range].

Abbreviations: BMI= body mass index; CAD= coronary artery disease; PCI= percutaneous coronary intervention; CABG= coronary artery bypass grafting; ACEi= angiotensin converting enzyme inhibitor; ARB= angiotensin receptor blocker; ECG= electrocardiogram; eGFR= estimated glomerular filtration rate

**Supplemental Table 4.** Findings on coronary computed tomography angiography in patients stratified by presence anginal symptoms and troponin concentration

|  | **Patients without anginal symptoms** | | | **Patients with anginal symptoms** | | | P-value* |
| --- | --- | --- | --- | --- | --- | --- | --- |
|  | **Overall** | **<5ng/L** | **5ng/L to**  **99^th^ centile** | **Overall** | **<5ng/L** | **5ng/L to**  **99^th^ centile** |  |
| **Number of participants** | 144 | 53 | 91 | 106 | 30 | 76 |  |
| **Stenosis severity** |  |  |  |  |  |  | 0.693 |
| Normal | 55 (38.2) | 29 (54.7) | 26 (28.6) | 39 (36.8) | 18 (60.0) | 21 (27.6) |  |
| Non-obstructive CAD | 52 (36.1) | 14 (26.4) | 38 (41.8) | 38 (35.8) | 6 (20.0) | 32 (42.1) |  |
| Mild (<50%) | 40 (27.8) | 9 (17.0) | 31 (34.1) | 25 (23.6) | 4 (13.3) | 21 (27.6) |  |
| Moderate (50-70%) | 12 (8.3) | 5 (9.4) | 7 (7.7) | 13 (12.3) | 2 (6.7) | 11 (14.5) |  |
| Obstructive CAD | 37 (25.7) | 10 (18.9) | 27 (29.7) | 29 (27.4) | 6 (20.0) | 23 (30.3) |  |
| One vessel | 20 (13.9) | 5 (9.4) | 15 (16.5) | 13 (12.3) | 1 (3.3) | 12 (15.8) |  |
| Two vessels | 10 (6.9) | 3 (5.7) | 7 (7.7) | 12 (11.3) | 5 (16.7) | 7 (9.2) |  |
| Three vessels | 7 (4.9) | 2 (3.8) | 5 (5.5) | 4 (3.8) | 0 (0.0) | 4 (5.3) |  |
| **Atherosclerotic burden** |  |  |  |  |  |  |  |
| Segment involvement score | 2.0 [0.0, 5.2] | 0.0 [0.0, 3.0] | 3.0 [0.0, 6.0] | 2.0 [0.0, 6.0] | 0.0 [0.0, 5.2] | 2.0 [0.0, 6.0] | 0.787 |
| Segment stenosis score | 2.0 [0.0, 7.2] | 0.0 [0.0, 3.0] | 3.0 [0.0, 10.0] | 2.0 [0.0, 8.0] | 0.0 [0.0, 7.8] | 3.0 [0.0, 8.0] | 0.692 |
| CT-Leaman score | 3.2 [0.0, 9.4] | 0.0 [0.0, 4.6] | 5.2 [0.0, 10.4] | 3.2 [0.0, 10.3] | 0.0 [0.0, 10.1] | 4.3 [0.0, 10.2] | 0.749 |

Presented as No. (%), mean (SD) or median [inter-quartile range].

Abbreviations: CAD= coronary artery disease; CCTA=coronary computed tomography angiography.

* P-value for comparison between patients with and without anginal symptoms.

**Supplemental Figure 1.** Flow diagram of the study population

**Supplemental Figure 2.** Adjusted odds ratio of coronary artery disease on coronary computed tomography angiography in patients with intermediate (between 5 ng/L and the sex-specific 99th centile) *versus* low troponin concentrations (<5 ng/L)

**Supplemental Figure 3.** Odds ratio of coronary artery disease on coronary computed tomography angiography in patients with intermediate (between 5 ng/L and the sex-specific 99th centile) *versus* low troponin concentrations (<5 ng/L) in sensitivity analysis restricted to those without known coronary artery disease

**Supplemental Figure 4.** Odds ratio of coronary artery disease on coronary computed tomography angiography in patients with anginal symptoms *versus* those with no anginal symptoms
